# Individual Pharmacotherapy Management (IPM) - I: a group-matched retrospective controlled clinical study on prevention of complicating delirium in the elderly trauma patients and identification of associated factors

**DOI:** 10.1186/s12877-021-02630-y

**Published:** 2022-01-06

**Authors:** Luise Drewas, Hassan Ghadir, Rüdiger Neef, Karl-Stefan Delank, Ursula Wolf

**Affiliations:** 1 Pharmacotherapy Management Department, University Hospital Halle (Saale), Ernst-Grube-Straße 40, 06120 Halle (Saale), Germany; 2 Internal Medicine Clinic I, Martha Maria Hospital, Halle (Saale), Germany; 3 Internal Medicine - Cardiology and Pulmonology, Asklepios Clinic Wandsbek, Hamburg, Germany; 4 Department of Orthopedics, Trauma and Reconstructive Surgery, Division of Geriatric Traumatology, University Hospital Halle (Saale), Halle (Saale), Germany; 5 Department of Orthopedics, Trauma and Reconstructive Surgery, University Hospital Halle (Saale), Halle (Saale), Germany

**Keywords:** Delirium, Elderly patients, Traumatology, Prevention, Polypharmacy, Medication review, Risk factors, Adverse drug reactions, Serotonin syndrome, ICD-classification

## Abstract

**Background:**

Delirium is one of the most frequent complications in hospitalized elderly patients with additional costs such as prolongation of hospital stays and institutionalization, with risk of reduced functional recovery, long-term cognitive impairment, and increased morbidity and mortality. We analyzed the effect of individual pharmacotherapy management (IPM) in the University Hospital Halle in geriatric trauma patients on complicating delirium and aimed to identify associated factors.

**Methods:**

In a retrospective controlled clinical study of 404 hospitalized trauma patients ≥70 years we compared the IPM intervention group (IG) with a control group (CG) before IPM implementation. Delirium was recorded from the hospital discharge letter. The medication review and data records included baseline data, all medications, diagnoses, electrocardiogram (ECG), laboratory and vital parameters during hospitalization. The IPM internist and the senior trauma physician guaranteed personnel and structural continuity in the implementation of the interdisciplinary patient rounds.

**Results:**

There was a highly matched congruence between CG and IG in terms of age, gender, residency, BMI, most diagnoses, and injury patterns to compare the two groups. The total number of medications per patient was 11.1 ± 4.9 (CG) versus 10.4 ± 3.6 (IG). Our targeted IPM focus on 6 frontline aspects with reduction of antipsychotics, anticholinergic burden, benzodiazepines, serotonergic opioids, elimination of pharmacokinetic and pharmacodynamic drug interactions and overdosage reduced complicating delirium from 5% to almost zero at 0.5%. The association of IPM with a significant 10-fold reduction, OR = 0.09 [95% CI 0.01–0.7], in univariable regression, maintained of clinical relevance in multivariable regression OR = 0.1 [95% CI 0.01–1.1]. Factors most strongly associated with complicating delirium in univariable regression were cognitive dysfunction, nursing home residency, muscle relaxants, antiparkinsonian agents, xanthines, transient disorientation documented in the fall risk scale, antibiotic-requiring infections, antifungals, antipsychotics, and intensive care stay, the two latter maintaining significance in multivariable regression.

**Conclusions:**

IPM is associated with a highly effective prevention of complicating delirium in the elderly trauma patients. For patient safety it should be integrated as an essential preventative contribution. The associated factors help identify patients at risk.

## Background

Delirium occurs in the general population with a prevalence of 1–2% [1]. However, it is found in 10–15% of elderly patients presenting to the emergency department [1], and delirium is one of the most frequent and feared complications in elderly patients during hospitalization [2, 3]. It causes significant additional economic and social costs such as prolongation of hospital stay by an average of 8 days, reduced functional recovery, long-term decline in cognitive performance, and increased morbidity and mortality [1–4]. Symptoms of delirium persist in one-third of patients, which is associated with a generally worse prognosis for patients [2, 5]. Delirium has therefore become an increasing focus of research and everyday life in recent years.

Delirium is considered to have a multifactorial genesis [1].

90% of patients > 65 years of age regularly take prescription medications. Of these, approximately 20–50% have an anticholinergic side effect profile [6]. In 12–39% of cases, medications were the sole determining cause for the development of delirium [7]. Polypharmacy, described in the metadata analysis by Masnoon et al. [8] as permanent use of at least 5 medications, increases the incidence of delirium by a factor of 4.5 in the elderly population [1, 9]. The most common drugs associated with delirium were benzodiazepines, opioids, and preparations with anticholinergic side effects such as antiemetics, spasmolytics, antiarrhythmics, antihistamines, corticosteroids, muscle relaxants, and psychotropic agents [1, 7].

Elderly patients are additionally more susceptible to adverse drug reactions (ADR). The blood-brain barrier becomes more permeable with age, and the renal and hepatic filtering and metabolizing processes deteriorate [10, 11]. Damage to the blood-brain barrier by postoperative inflammatory processes [12] with an increase in inflammatory parameters such as Interleucin-6 and Interleucin-8 is discussed as a further cause of delirium [13, 14].

Another increase in the incidence of delirium is seen in acute inpatient admissions and traumatology surgical treatments. For example, the incidence of delirium in patients undergoing orthopedic and trauma surgical treatment ranges from 12 to 51% according to US data [1]. These figures are consistent with intervention studies from Germany. Here, 20.2–20.8% of > 70-year-old patients developed delirium after general surgical care [15]. In 2008, Robinson et al. compared the delirium incidences of different surgical procedures and concluded that the degree of surgical exposure was related to the development of delirium. They found that cataract surgery caused only low levels of surgical stress, as the incidence of delirium was 4%, whereas high-risk surgeries, such as vascular procedures, had an incidence of 36% [16].

In the decades-long efforts to explain delirium, it was initially regarded as an agitation disorder with alteration of consciousness, i.e., as a mere mental state problem [1]. A clear terminology was lacking.

Z. J. Lipowski described this problem as “semantic confusion” in 1983. He postulated that scientific work in the field of delirium was hindered by the lack of a clear definition and the absence of precise diagnostic criteria [17]. At that time, “acute state of confusion”, “senile delirium”, “acute brain syndrome” or “pseudosenility”, among others, were used in parallel. Lipowski first proposed to use the term “delirium” exclusively for transient, global cognitive disorders, joining the Diagnostic and Statistical Manual of Mental Disorders of the American Psychiatric Association (DSM)-III classification system updated in 1980 [17].

This work was the first to distinguish between “hyperactive” and “hypoactive” delirium [18]. Since then, 4 motor subtypes have been described. Common to all 4 subtypes are altered sleep-wake rhythms, disturbances in attention and memory, alterations in thinking and speech, and perceptual disturbances [19, 20].

The American DSM-5 currently describes delirium as a fluctuating disorder of attention and consciousness that develops acutely over hours to a few days. In addition, there is an alteration of cognition (disorientation, memory deficit, disturbances in perceptual ability). These disturbances cannot be explained by neurocognitive disorders, such as dementia. Instead, there is evidence from the medical history or from clinical examinations that this disorder is a direct consequence of a medical condition, intoxication, or withdrawal [21]. This is inconsistent with the current International Classification of Diseases (ICD)-10 classification of delirium. There, in its main heading it is described as an “aetiologically non-specific brain-organic syndrome not caused by alcohol or other psychotropic substances, characterized by simultaneous disturbances of consciousness on the one hand and at least two of the following disturbances on the other: disturbances of attention, perception, thinking, memory, psychomotor function, emotionality or sleep-wake rhythm. The duration varies greatly [...]” [22].

The aim of the present study was to investigate the intervention effect of individual pharmacotherapy management (IPM) in trauma geriatric patients with subsequent medication adjustment on the incidence of complicating delirium and to identify predisposing associated factors. The hypothesis is that because some are medication-induced, a certain proportion is preventable by targeted clinical pharmacological intervention in perioperative medication.

## Methods

### Study design, patient population and setting

**Fig. 1:**
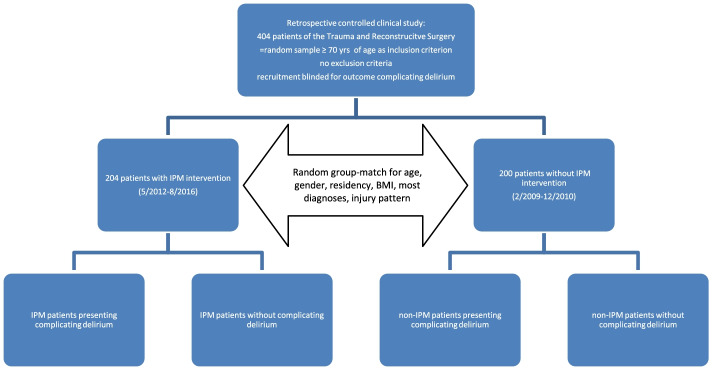
Design of the retrospective controlled clinical study, patient recruitment and group-matched confounders

To investigate the effects of IPM on delirium, we conducted a retrospective controlled clinical study enrolling 404 inpatients ≥70 years of age from the Department of Trauma and Reconstructive Surgery for a two-arm evaluation: 204 patients with IPM intervention from the IPM period and 200 patients without IPM from the period before IPM implementation. Completion of random recruitment, blinded for the outcome complicating delirium, provided two inpatient samples that showed group-matching for age, gender, residency, BMI, most diagnoses, and injury patterns, which were thus excluded as important potential confounders of delirium manifestation (Fig. 1).

IPM for inpatients ≥70 years of age at the Department of Trauma Surgery at the University Hospital Halle (Saale) (UKH) started in February 2011 with the implementation of an interdisciplinary fortnightly ward rounds on the traumatology ward in addition to individual medication reviews. Uniformity of patient rounds was ensured by the continuous presence of the same responsible internal medicine/pharmacotherapy management specialist with the same senior geriatric traumatology physician, accompanied by residents and geriatricians, medical students, and nurses. From the intervention period, 204 patients ≥70 years of age (intervention group, IG) were enrolled with samples from May 2012 to August 2016. The control group (CG) included 200 patients ≥70 years of age who were hospitalized in the same ward between February 2009 and December 2010. Because outcomes had already occurred at the time of recruitment, both cohorts included samples that were blinded to outcome. In the Department of Trauma Surgery, the total number of geriatric patients aged ≥70 years admitted as inpatients in the years of recruitment was: 335 patients in 2009 and 459 patients in 2010, 433 patients in 2012, 477 in 2013, 471 in 2014, 428 in 2015, and 431 in 2016. Recruitment of patients in the intervention group over the years was intended to assess lasting and stable potential effects. The patient recruitment in each group and from the different years was at random and performed individually for each group. The final group population was a matched one comparing control and intervention group with respect to age, gender, residency, BMI, most diagnoses, and injury patterns. This group-match resulted by chance and it was not proactively controlled in either group during the recruitment phase. For the conductance and the interpretation of the study results, the agreement of the groups with respect to relevant confounders is one of the key conditions met in order to adequately compare the two groups excluding decisive confounders for outcome.

There were no relevant changes in perioperative medical or nursing management over time. The spectrum of trauma and fractures also remained almost constant, and there were no discernible changes relevant to the analysis. Since being employed in the Department of Trauma Surgery at the University Hospital Halle and in particular since his position as senior physician in this from 2008 onwards, always this same senior physician in geriatric traumatology has been acting as a contributing team leader in terms of a multimodal delirium prevention involving physicians and nursing staff of the ward with established prevention approaches since then. This applies equally to the control and intervention groups. Since 2008, throughout the entire survey period including both, CG and IG, delirium prevention remained a multimodal approach that consistently focused on reorientation, optimized hydration, early mobilization, appropriate pain management, and early diagnosis for timely treatment of infection. IPM implementation started on an ongoing basis in 2011 as a step-up to optimize care and minimize further or repeated risk for these elderly and oldest-old patients from polypharmacy or even single medical agents. The multimodal preventive approach before and alongside IPM was not declared non-pharmacological, as in terms of early intervention for infections, this aspect was always integrated.

### IPM intervention

**Fig. 2:**
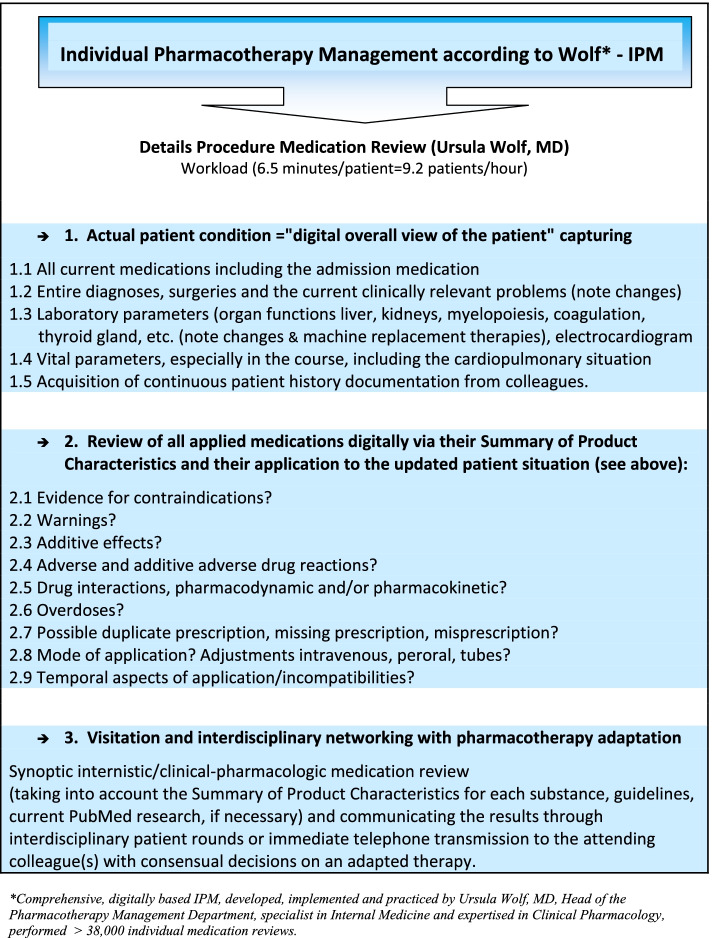
IPM procedure after Wolf*

The standardized, reproducible three-stage procedure of the implemented IPM (Fig. 2) always synoptically considers internistic and clinical-pharmacologic aspects due to the professional qualification and expertise of the responsible physician. IPM is based on the fully digitalized patient medical record by a hospital information system software (Orbis system) in the clinical environment. It provides the medication reviewer with a comprehensive “view” of the individual patient with his/her medication list, diagnoses, surgeries, updated laboratory (organ functions) and vital signs, ECG (Table 1) and ongoing clinical documentation from colleagues. The resulting IPM after Wolf (Fig. 2) takes into account the drug-specific professional information of all medications, medical guidelines and, if necessary, further clarifying updated PubMed searches. Indications and contraindications, warnings, additive effects, adverse drug reactions, pharmacodynamic and/or pharmacokinetic drug interactions, overdoses, duplicate prescriptions, missing prescriptions, erroneous prescriptions, temporal aspects of use/application/incompatibilities are recorded and reviewed. Finally, for all patients visited, individual therapy recommendations are implemented immediately after presentation of identified risks from drug effects, ADR, and drug interactions in an interdisciplinary consensus. Both, the further drug regimen and the therapy changes made are communicated as recommendations to the patient’s physician providing outpatient treatment.

**Table 1:** Variables collected for data analysis and included in the individual medication review

**• Demographics:** age, gender, type of residence (home/nursing home)	
**• Vital parameters at admission:** BMI, blood pressure (day course), heart rate (day course)	
**• Continuous and acute medication:** number of drugs, Angiotensin converting enzyme inhibitors (ACE inhibitors), sartans, calcium antagonists, differentiated ß-blockers, α-blockers, antibiotics, antifungals, antiarrhythmics, antidementives, anticonvulsants, different oral anticoagulants, bisphosphonates, different antiplatelet drugs, different diuretics, antipsychotics, antidepressants, St. John’s wort, oral antidiabetics, insulin, antiparkinsonian drugs, benzodiazepines, proton pump inhibitors (PPI) (incl. dosage), ophthalmics, urological drugs, muscle relaxants, opioids, “Würzburger pain drip“^a^, tramadol, nonsteroidal anti-inflammatory drugs (NSAIDs), further analgesic agents, antiemetics, thyroid hormones, xanthines, uricosurics, uricostats, statins, vitamin D, corticosteroids, other drugs (e.g. hormones, cytostatics)	
**• Laboratory parameters at admission:** blood count, electrolytes, inflammation parameters, renal function parameters during course of stay, myoglobin, coagulation parameters, urinalysis	
**• ECG (if available online):** rhythm, frequency, QT interval^b^, atrioventricular block (AV block)	
**• Diagnoses**^c^**:** arterial hypertension, heart failure, severe delirium, cognitive impairment to dementia, Parkinson’s disease, further central nervous system (CNS) disorders, chronic obstructive pulmonary disease (COPD), diabetes mellitus, osteoporosis, chronic kidney disease	
**• Additional course aspects:** changes in laboratory findings, blood pressure, heart rate, temperature, cognitive changes/disturbances, pain symptoms and profile, other subjective complaints of the patient	
**• Other parameters:** acute admission injury, operation, transient stay in IMC^d^ or ICU^e^, hemodialysis, length of hospital stay, perioperative infections, fall risk scale according to Huhn (0–31 points, broken down according to: age, mental status, excretion, history of falls, gait/balance, activities, medication, alcohol), pacemaker, defibrillator, infections requiring antibiotics, contrast medium application	

^a^Combination of tramadol, metamizole, and metoclopramide administered intravenously or partially orally

^b^time from the start of the Q wave to the end of the T wave (measurement on ECG)

^c^coded in the hospital discharge letter

^d^Intermediate care

^e^Intensive care unit

### Data collection

We focussed on the outcome “complicating delirium“. The defined “complicating delirium” is our clinical definition to select and determine the most robust manifestation of delirium that as a consequence is consistently documented in the patient hospital discharge letter. The discharge letter includes all diagnoses and information on the inpatient pre-, peri-, and post-operative course with reference to any kind of adverse or unexpected events or complications. To date, there are no references or definitions in the literature for severe delirium. “Complicating delirium “was defined as a delirium necessitating further investigations as laboratory parameters, cranial computed tomography or magnetic resonance imaging, and/or psychiatric consultation. In the context of the diagnostic assessment of the complicated clinical delirium situation, the described consecutive examinations were protocolized in the discharge letter as an additionally indicated diagnostic procedure to further investigate the patient delirious status. Complicating delirium simultanesously encompasses both, the criteria of the DSM-4 as well as the current DSM-5; among others, the presence of disorders of attention and consciousness and at least one other cognitive deficit developed over hours or a few days [21]; and it includes hyperactive, hypoactive, and mixed subtypes. For the anonymously sampled 404 patients, a total of 115 parameters (if available online) were recorded from the digital hospital information system Orbis (Table 1). Medication data were collected from the most comprehensive medication list during the patient’s hospitalization, thus including transient antibiotics.

### Literature search

The literature search was conducted online via PubMed - NCBI from November 2016 to January 2021. To obtain a comprehensive overview of recent national and international studies, we used the following search terms: ‘delirium in elderly people’, ‘drug-induced delirium’, ‘delirium polypharmacy’, ‘delirium post surgery elderly’, ‘postoperative delirium in the elderly’, ‘delirium definition’, ‘delirium postoperative elderly epidemiology’, ‘postoperative delirium prevention’, ‘delirium medication review’, ‘delirium medication analysis’, ‘delirium prevention’. We applied the filter “Species: Humans.” Literature management and citation was done with Citavi version 5.5.0.1.

### Statistical analysis

Statistical analysis was supervised by the consulted Institute of Medical Epidemiology, Biometry, and Informatics. We used Microsoft Excel 2016 for anonymous data collection and SPSS Statistics 24 for data analysis. After descriptive analysis of the two groups and univariable logistic regression in relation to complicating delirium, we performed multivariable regression analysis for all variables with a *p* value ≤0.05 to measure the association with this outcome, adjusting for confounders and using a 95% confidence interval (CI) to correct for multiple testing. We performed logistic regression analysis including the entire study population to identify independent factors associated with complicating delirium.

## Results

**Fig. 3:**
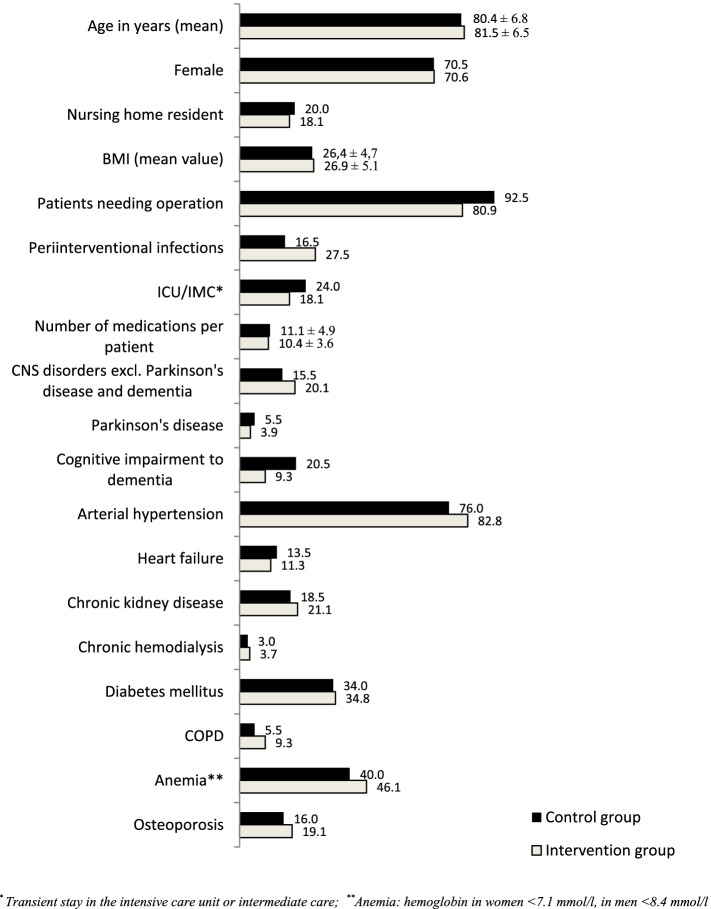
Baseline data and diagnoses comparing control and intervention group (Percentage prevalence numbers (%) except mean values ± standard deviation (SD) for age, BMI, and number of medications)

**Fig. 4:**
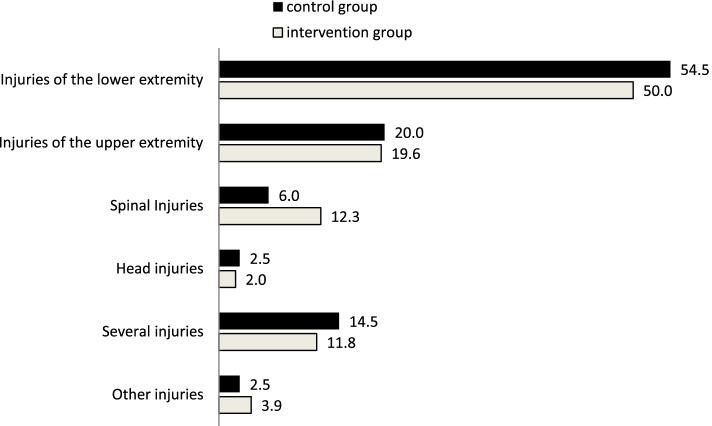
Injury pattern (%) of geriatric patients admitted to the traumatology department comparing control and intervention group

Descriptive analysis of the IG (*n* = 204 patients) and the CG (*n* = 200 patients) in terms of baseline data as age, gender, residency, BMI (Fig. 3) and injuries leading to admission (Fig. 4) showed a comparable distribution pattern over the entire observation period.

In both groups, the trauma patients were almost oldest-old and predominantly female (71% in each group) (Fig. 3). Most patients (80%) lived in their own homes. More than 80% of patients in both groups required surgical intervention. The similar number of prescribed medications averaged 11.1 ± 4.9 in the CG and 10.4 ± 3.6 in the IG. The two groups differed only slightly in the distribution pattern of concomitant diagnoses (Fig. 3). Pre-existing central nervous system (CNS) diseases, excluding Parkinson’s disease and cognitive impairment to dementia, were slightly more frequent in the IG, less frequent cognitive impairment. The prevalence of AV block I-III in the 80 available ECGs was the same in both groups (21%). Laboratory parameters analyzed included hemoglobin content and glomerular filtration rate (according to the Berlin Initiative Study eGFR equations (BIS formula)), leukocytes, mean corpuscular volume (MCV), and serum sodium. In addition to the similar prevalence of anemia in both groups (Fig. 3), increased MCV as a possible indication of vitamin B12 and/or folic acid deficiency anemia was equally distributed in macrocytic anemia, and the same was true for microcytic hypochromic anemia. Leukocytosis was found in 42% of patients in CG and 46.6% in IG. A glomerular filtration rate (GFR) G3 according to KDIGO (Kidney Disease: Improving Global Outcomes) was present in 53% in the CG and 58.3% in the IG. Only one-third of patients in both groups had normal renal function. The prevalence of chronic kidney disease shown in Fig. 3 can be further broken down according to the GFR determined here by BIS. There was no difference between the control and intervention groups in terms of different severity levels. Hyponatremia present at admission was found in both patient groups with CG 6.5% and IG 10.3%. Regarding blood pressure values measured during the course of a hospital day, 18.5% of patients in the CG and 12.8% in the IG had hypotensive blood pressure values (systolic < 120 mmHg).

Primarily, 50% of patients in both groups were affected by traumatologic injuries to the lower extremity **(**Fig. 4**).** Only the prevalence of spinal injuries was higher in the IG. Injuries to the upper extremity, head, or several concurrent injuries on admission were almost equally distributed.

**Fig. 5:**
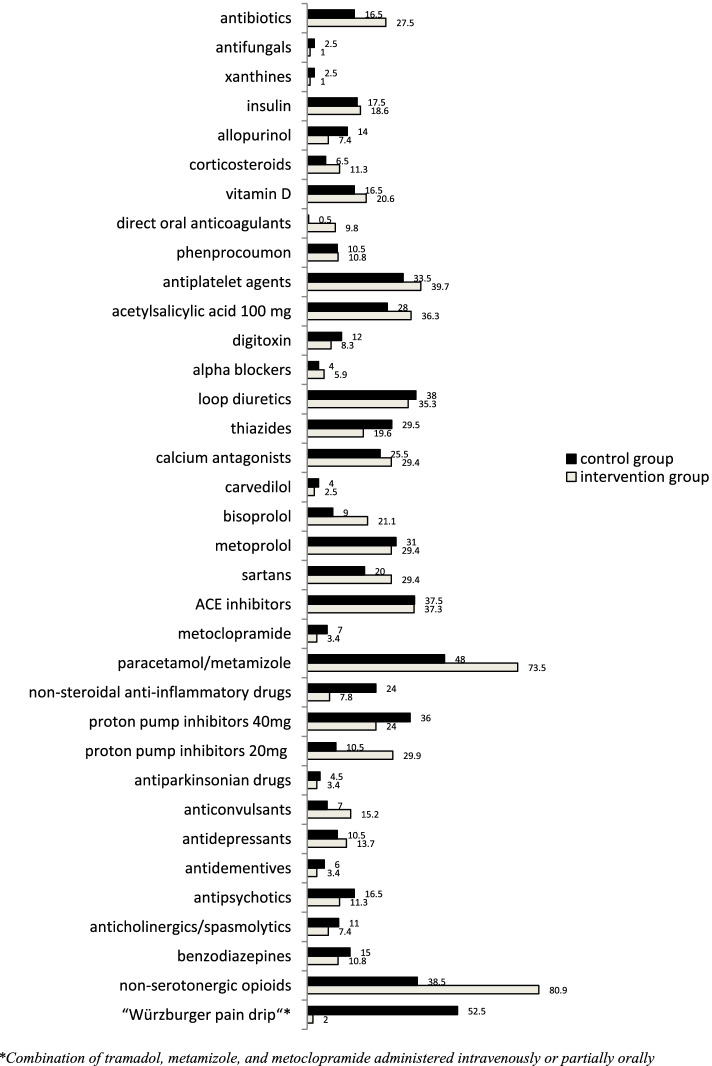
Percentage prescription rates (%) of drugs and drug groups comparing control and intervention group

The individual medication data analyses focused on all different drug groups and we compared the distribution frequencies of the entire perioperative medications of both groups (Fig. 5).

In particular, differences in the prescription rate of medications switched by the IPM were evident. The “Würzburger pain drip” was almost withdrawn from 52,2 to 2.0% in the IG. NSADs, metoclopramide, and benzodiazepines were reduced in the IG. In contrast, to compensate for this, prescription of non-serotonergic opioids, as well as paracetamol, and metamizole increased in the IG. Thiazides and allopurinol were reduced in IG. Consistent with their more recent approval, direct oral anticoagulants predominated in IG. Within the PPI doses distributions, the IG reduction of inadequately high PPI doses from 40 to 20 mg stands out as an adapted adequate and only transient prophylactic dose in the perioperative setting. Antibiotics were applied more frequently in IG (27.5%) than in CG (16.5%) (Fig. 5).

Due to the short inpatient length of stay in the perioperative setting and the fact that many drugs require gradual deprescribing approach, the therapeutic effect of the drug therapy recommendations of the IPM could only be partially captured. In addition, we did not consider the frequent approach of dose reductions in this data analysis.

**Fig. 6:**
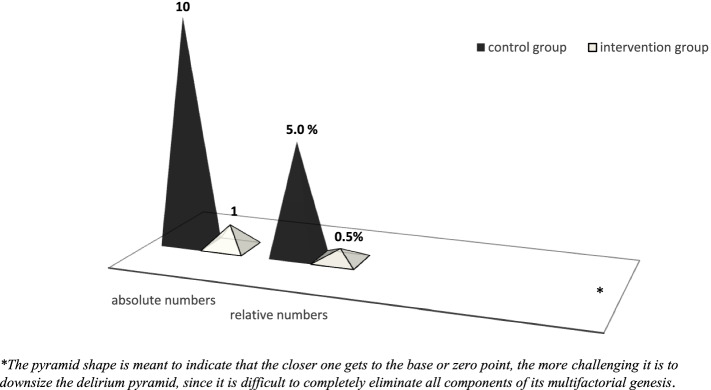
Incidence of complicating delirium comparing control (*n* = 200 patients) and intervention group (*n* = 204 patients)

Complicating delirium manifested in 5% of patients in the CG (10 of 200 patients) and 0.5% in IG (1 of 204 patients) (Fig. 6).

Affected patients in CG were predominantly male (8.5% of 59 men versus 3.5% of 141 women).

In IG, IPM reduced the risk of complicating delirium by 90.2%. Gender distribution was the same in both groups.

Univariable logistic regression from all variables revealed the following confounders with clinically relevant association with complicating delirium, defined with a strong odds ratio (OR) ≥ 2 or ≤ 0.5 (Table 2).

**Table 2:** Variables showing a clinically relevant association (defined as a strong OR ≥ 2 or OR ≤ 0,5) with complicating delirium (univariable regression analysis including all patients *n* = 404)

	*P*-value	Odds ratio	95% Confidence interval	Total number
IPM	0.03	**0.09**	0.01–0.7	204
Cognitive impairment to dementia	0.001	**9.5**	2.7–33.5	68
Nursing home resident	0.001	**8.1**	2.3–28.3	77
Intensive care stay	0.012	**4.8**	1.4–16.0	85
Anemia	0.17	**2.4**	0.7–8.1	174
BMI ≤20 kg/m2	0.4	**2.5**	0.3–21.5	19 *(m*^*1*^*62)*
COPD	0.2	**2.9**	0.6–14.1	30
Diabetes mellitus	0.2	**2.3**	0.7–7.8	139
Infection requiring antibiotics	0.003	**6.6**	1.9–23.2	89
Fall in hospital	0.2	**2.8**	0.5–13.5	31
Men	0.2	**2.0**	0.6–6.8	119
Parkinson’s disease	0.05	**4.9**	1.0–24.5	19
Chronic hemodialysis	0.3	**3.2**	0.4–26.8	13
Atrial fibrillation	0.05	**12.0**	1.0–143.9	13 *(m*^*1*^*324)*
Antipsychotics	0.001	**12.3**	3.5–43.5	56
NSAIDs	0.3	**2.0**	0.5–7.9	64
Paracetamol / Metamizole	0.2	**3.0**	0.6–13.9	246
Muscle relaxants	0.2	**4.8**	0.5–42.2	9
Digitalis	0.4	**2.0**	0.4–9.7	41
Corticosteroids	0.3	**2.3**	0.5–11.3	36
Antifungal drugs	0.09	**6.5**	0.7–58.7	7
Memantine	0.4	**2.5**	0.3–20.8	16
Antiparkinsonian drugs	0.03	**6.0**	1.2–30.5	16
Xanthines	0.002	**17.2**	2.9–101.0	7
Fall risk scale according to Huhn				394 *(m*^*1*^*10)*
temporarily disoriented	0.006	**6.7**	1.7–26.1	41
permanently disoriented	0.1	**3.6**	0.7–19.0	37
occasional alcohol consumption	0.1	**2.9**	0.7–11.4	46
restrictions in mobility	0.2	**3.7**	0.5–29.5	136
bladder catheter / enterostoma	0.1	**3.6**	0.6–19.9	90
bladder / stool incontinent	0.2	**3.9**	0.5–28.9	41

m^1^ missing values

A relevant association with a strong OR ≥ 2 was found with both preventable factors such as in-hospital falls, inserted urinary bladder catheter, anemia, body mass index (BMI) ≤ 20 kg/m^2^, dementia, and various medications, and with invariant or less variable factors such as gender, chronic hemodialysis, nursing home residency, and Parkinson’s disease. Items from Huhn’s fall risk scale (collected by nursing staff on inpatient admission) such as temporary or permanent disorientation, bladder catheter or enterostoma, incontinence, occasional alcohol consumption were associated with delirium. Other less pronounced associations (OR < 2) were seen with: Hyponatremia, type of injury on admission, leukocytosis, benzodiazepines, metoclopramide, tramadol, and “Würzburger pain drip“, the latter with an OR = 1.68 [95% CI 0.36–7.91].

Multivariable regression analysis was performed with all confounders revealing an association with *p*-value ≤0.05 and is shown in Table 3. To achieve sufficient test power with a patient number of 404, the variable “atrial fibrillation” was excluded due to the small number (*n* = 80) of digitally available ECGs. The resulting OR > 1 after all adjusted variables indicate that there remains an independent increased association of the variable with the occurrence of complicating delirium. Only IPM was associated with an independent tenfold risk reduction for complicating delirium, according to the multivariable model.

**Table 3:** Measures of association with complicating delirium from multivariable regression analysis including variables with *p*-value ≤0.05 from univariable regression

	*P*-value	Odds-ratio	95% Confidence interval	Number
IPM	0.06	**0.1**	0.01–1.1	204
Cognitive impairment to dementia	0.3	**2.8**	0.3–22.9	68
Nursing home resident	0.3	**2.7**	0.4–17.5	77
Intensive care stay	0.05	**5.2**	1.0–27.1	85
Infection requiring antibiotics	0.1	**3.5**	0.7–18.4	89
Antipsychotics	0.01	**8.2**	1.6–42.6	56
Muscle relaxants	0.2	**15.6**	0.3–728.7	9
Antiparkinsonian drugs	0.5	**2.4**	0.2–30.3	16
Xanthines	0.05	**11.8**	1.0–132.8	7
Antifungal drugs	0.7	**1.9**	0.1–34.9	7
Fall risk scale according to Huhn - Temporarily disoriented	0.5	**1.7**	0.3–9.9	41

## Discussion

The study documents a high association of the applied IPM as a synopsis of internal medicine and clinical pharmacology with the reduction of complicating delirium in geriatric trauma patients. Still underrepresented in previously published study data, it potentially contributes as a preventive tool to improve patient care and patient safety in a clinically relevant way. Our data additionally confirm further and partly established factors associated with increased incidence of complicating delirium, including special drug groups.

The incidence of complicating delirium, 5% in the control group, is lower than in figures from the current literature, as the latter mostly include the entire spectrum of delirium**.** They range from 12 to 51% in different studies [1, 9, 15, 23, 24]. The large discrepancy between these literature studies can be partly explained by the different assessment of delirium and the difficulties in adequately recognizing it in extent, especially for the hypoactive form. Therefore, we focused on a more robust form of delirium for comparison, namely the concise manifestations complicating hospitalization as documented in the hospital discharge letter, although it is reasonable to assume that there is a higher incidence of noncomplicating or less pronounced manifestation of delirium after traumatology procedures. However, the certainty of an impressive reduction in the robust form of our defined complicating delirium by IPM remains, as possible documentation deficits would have to be assumed with continuity of responsibilities in CG and IG alike. In addition, the briefing of the staff and the detailed self-history we apply, as well as the patient’s history by relatives or others, help as an important prerequisite to distinguish dementia from delirium from the outset. Because different subtypes of delirium are often less accurately documented, we must be aware of a higher incidence of clinically less prominent delirium, which was purposefully not included in our definition of complicating delirium for either group because of the assumed uncertain documentation. Meagher et al. [25] applied three assessments for delirium subtype identification in their longitudinal study of 100 palliative care patients: the Delirium Motor Subtype Scale (DMSS), the Delirium Rating Scale-Revised-98 (DRS-R98), and the Cognitive Test for Delirium (CTD). Delirium phenomenology was stable during delirium episodes in 62%. Subtypes differed in non-cognitive symptoms but not in cognitive subscale scores. The latter should therefore be preferred to identify less obvious manifestations [25]. The possibility of a longitudinal study design on ongoing episodes of delirium in the palliative care may reflect its typical socalled the “best supportive care “(BSC) situation resulting from its inherently frequent application of combined psychotropic drugs, sedatives, and analgesics with resulting ADR and pharmacodynamic and pharmacokinetic interactions. Moreover, the palliative care group also frequently suffers from organ dysfunction that slows the capacity of drug metabolism and renal elimination, so attention must be paid to compensatory drug dose adjustment. Focusing on dose adjustment in relation to laboratory data on organ function has been a common process within IPM that is not further quantified here. This essentially requires knowledge of the exact drug metabolism/excretion in the human body from its drug-specific professional information as a profound basis of applied IPM.

The tenfold reduction of complicating delirium in association with IPM is clinically relevant. This is also related to the consistent implementation of IPM over years including a complete restructuring of the drug pain management. Withdrawal of tramadol and other serotonergic opioids proved to be an important step in this process. Presumably, there is an unconsidered overlap with serotonin syndrome in the diagnosis of delirium, as elderly patients often take serotonergic agents, e.g., from the broad spectrum of antidepressants, which should not be combined with serotonergic opioids.

On average, the number of preset medications was similar in both groups, and the need for perioperative analgesics and possibly antibiotics cannot be excluded. The common finding from the international literature is that medications and polypharmacy can promote or even trigger delirium [1, 7, 16, 26].

Masnoon et al. found 138 definitions of polypharmacy, a large proportion of which were simply quantification of medications taken [8]. Only 6.4% of the articles distinguished between adequate and inadequate polypharmacy. However, they also pointed out that a purely numerical definition makes it difficult to ensure safety and appropriateness in clinical practice. This is consistent with our data. The approximately equal number of medications taken in the control and intervention group suggest that, as described by Masnoon et al., it is not only the amount of medication that contributes to the development of delirium [8]. Moreover, medication of elderly and often multimorbid patients should be regularly and critically reviewed, as performed in a very comprehensive way by the presented IPM procedure, with additional focus on elimination of drug interactions, overdosage, attention to warnings and ADR often even cumulative.

In this context, early withdrawal of the “Würzburger pain drip” consisting of a fixed combination of tramadol, metoclopramide, and metamizole was an essential step of IPM with a potential “superior” long-term class effect. Some of these drugs have a high potential for interaction with synergistic ADR and even reciprocal influence on enzymatic metabolism and excretion rate. Metamizole can lead to acute deterioration of renal function and even acute renal failure [27]. Metoclopramide acts through its dopamine receptor-blocking mechanisms in the CNS. Even a single dose can lead to extrapyramidal symptoms and, in combination with antipsychotics, can cause malignant neuroleptic syndrome. Intravenous administration in elderly patients with existing conduction disorders, uncorrected electrolyte shifts or bradycardia promoted QT prolongation, AV block, sinus arrest, torsade de pointes, and cardiac arrest [28]. Tramadol exerts its analgesic effect through opioid, serotonergic and noradrenergic receptors [29]. It can cause hallucinations, confusion, and changes in cognitive and sensory performance, leading to the development and increased incidence of delirium [30].

Tramadol additionally accumulates in renal insufficiency and requires early adaption, which is often overlooked. Both, tramadol and metoclopramide are substrates of the highly polymorphic monooxygenase cytochrome P450 2D6 (CYP2D6), which has a high affinity and low capacity for its substrates, resulting in reduced drug efficacy especially when prodrugs such as tramadol must be activated by CYP2D6. Approximately 20–25% of all drugs used are metabolized by CYP2D6, but nearly 50% of all drugs used in the clinical setting (including antidepressants, antiemetics, beta-blockers, psychotropic drugs, and opioids) undergo this metabolism. In addition, the pharmacogenics regarding CYP2D6 polymorphism influences drug levels. It must be taken into account that undetected poor metabolizers account for 5–10% and 5% of all Western Europeans are considered CYP2D6 ultrarapid metabolizers [31, 32]. Switching from intravenous “Würzburger drip pain” to preferential peroral or subcutaneous administration of hydromorphone or tilidine/naloxone retard has been part of the successful reduction of complicating delirium. Tilidine is preferable to tramadol as a pure opioid agonist with greater analgesic potency and especially regarding psychiatric symptoms. The sustained-release form ensures uniform analgesia over 12 h, and impaired renal function does not lead to accumulation of pharmacologically active metabolites.

In hepatic impairment, the maximum plasma concentration of the active metabolite nortilidine is lower and the half-life is prolonged. It is subject to a first-pass mechanism and is metabolized by cytochrome P450 3A4 (CYP3A4) and cytochrome P450 2C19 (CYP2C19), among others, so restriction with potent analog inhibitors is required. Concomitant administration with serotonergic drugs may increase the risk of serotonin syndrome [33]. 

The unretarded form of hydromorphone, a μ-selective, pure opioid agonist, has been used to break through pain peaks. Because there is no opioid without an ADR, it can occasionally cause agitation, depression, euphoria, hallucinations, and nightmares [34]. Perioperatively we primarily use the sustained-release hydromorphone as a basic analgesic in combination with the unretarded form as an on-demand medication. After a few days postoperatively and especially at discharge, we de-escalate to tilidine. Only for minor injuries and minor surgical procedures do we start with tilidine from the beginning.

 In univariable observation, we found other factors associated with complicating delirium. This is reflected analogously in the various studies on delirium. In their metadata analysis of 10 prospective observational studies on preoperative risk factors, Oh et al. describe cognitive dysfunction as a relevant influencing factor for delirium [26]. Other studies also identify cognitive dysfunction as an important associated factor [1, 15, 35].

A BMI < 20 kg/m^2^ is documented as an influencing factor by Oh et al. [26] and Juliebo et al. [35]. There are further risk factors for delirium that are consistent with the association results of our study. These include nursing home residency [26], infections [1, 2], male gender [5], Parkinson’s disease [9], and antipsychotics [23].

Algiakrishnan et al. [7] detected an influence of various drugs on delirium, which was also found in our analysis: NSAIDs, muscle relaxants, digitalis, glucocorticoids, xanthines and antiparkinsonian drugs, benzodiazepines, and antiemetics. However, the total number of some drug groups in our cohorts is rather low. While both Raats et al. [36] and Myint et al. [37] found no association between preoperative anemia and postoperative delirium, our data showed at least a clinically relevant association with anemia with a more than doubled OR. The included age group of our patient collective was ≥70 years, and on average, patients in both groups were almost oldest-old at approximately 80 years. Oh et al. [26] could find six studies in which age showed a univariable association with delirium and two studies in which there was a significant association with the development of delirium after adjustment in the multivariable model. However, in the present study, no relevant association was found for age and delirium. The discrepancy may be partly due to the continuing difficulty in defining “age.” Many studies refer only to “old age” or the “elderly patient” [10, 26, 38]. Following the Lancet article by Beard et al. on the first World Health Organization (WHO) World Report on Aging and Health, old age/frailty may be seen as the progressive decline of physiological systems leading to increased vulnerability to stressors, resulting in negative risks such as need for care and death [39]. According to Beard et al., it is more an individual aspect and depends, for example, on socioeconomic status, among other factors, and also increases with numerical age. They postulate that the state of health in old age should not be determined by the presence or absence of disease, but should focus on the individual’s ability to function. The problem of complex definition is also evident in German-speaking countries; the widely used potentially inappropriate medication in the elderly (Priscus) list for assessing inadequate medication in elderly patients does not provide a definition for “elderly patients.” An important negative aspect of tools to improve polypharmacy in the elderly, such as the Medication Appropriateness Index (MAI), the Beers list or the Priscus list, the Fit fOR The Aged (FORTA) and the Screening Tool of Older Persons’ Prescriptions - Screening Tool to Alert to Right Treatment (STOPP-START), is that they can never capture the complex individual polypharmacy situation with drug interactions and organ functions in multimorbidity, as ensured by IPM. This may be the main reason for the very successful effect of “IPM according to Wolf”, which has not been achieved by any other intervention or strategy so far. 

Multivariable regression showed an OR = 5.2 [95% CI 1.0–27.1], *p* = 0.05, for an ICU/IMC stay, several studies confirm the independent impact of ICU stay on the development of delirium [40, 41]. Galyfos et al. analyzed 9 studies and described this as one of the most important associated factors with an OR = 6.12 [95% CI 4.7–7.9] [42].

Regarding blood pressure measured during the day course in hospital, 18.5% of patients had hypotensive blood pressure values (systolic < 120 mmHg) in the CG and 12.8% in the IG, but no relevant association with delirium emerged from univariable regression. Studies on the impact of perioperative hypotension are controversial. Wesselink et al. could not find a significant correlation between intraoperative hypotension and the development of delirium in on-pump cardiac surgery [43]. Hirsch et al. were able to establish an association between increased blood pressure fluctuations and the development of delirium during noncardiac surgery, but none with hypotension [44]. Nguyen et al. found a relationship between the development of delirium and low diastolic blood pressures (< 60 mmHg) in shock patients [45].

The higher preference of antibiotics in the intervention group is probably a consequence of the more numerous positive bacterial evidence in urinalysis.

Overall, 46.5% of patients in the CG and 53.9% in the IG were taking preset proton pump inhibitors. The inadequate and widespread prescribing of PPI in uncontrolled overdose appears to be related in part to the increased prescribing of direct oral anticoagulants and the increasing rate of use of antiplatelet agents as intended prophylaxis for gastrointestinal bleeding. With a concerning registration of predominantly preset therapeutic dosages of 40 mg daily rather than 20 mg prophylactically, our IPM aimed to adequately reduce the dosage of, for example, pantoprazole and omeprazole from 40 to 20 mg daily for perioperative stress ulcer prophylaxis. This IPM measure resulted in a deliberate reversal of the distribution frequency of the individual PPI dose favoring the adequate lower 20 mg in IG.

According to their ADR or misindication in more advanced renal dysfunction in 2/3 of the patients thiazides and allopurinol were deprescribed in IG. For chronic hemodialysis, an association was found in the univariable model, but since only 13 of the 404 patients were chronically dialyzed, it can only be evaluated to a very limited extent, as seen from the 95%CI (OR = 3.2 [95% CI 0.4–26.8], *p* = 0.3).

Hyponatremia at the time of admission was found in both patient groups, CG 6.5% and IG 10.3%, but no association measure with complicating delirium. Wang et al. detected a correlation between postoperative hyponatremia and postoperative delirium after orthopedic surgery with an OR of 3.0 [46].

Interestingly, in the univariable regression, several items from the Huhn risk fall scale assessed by nurses during inpatient admission, such as temporary or permanent disorientation, bladder catheter or enterostomy, limitations in mobility, incontinence, and occasional alcohol consumption were associated with delirium. Approximately 1/3 of patients described as cognitively impaired preoperatively develop postoperative delirium and have up to a 2-fold increased risk of mortality [1, 26, 47]. Cognitive impairment to dementia was analogously associated with complicating delirium in our study, with a clinically relevant OR of 2.8 in multivariable regression analysis.

Transient disorientation showed an OR almost twice as high compared with permanent disorientation, possibly indicating already mild manifestations or precursors of delirium. Regarding alcohol consumption, a urinary bladder catheter, and postoperative delirium, several studies [1, 7, 9, 40] documented an association. Brouquet et al. described a mobility restriction with > 20 s lasting “timed get up and go” as a significantly associated factor [48].

Delirium prevention by medication review is an underrepresented approach. A 2019 Cochrane review of delirium prevention interventions in the elderly in institutional long-term care by Woodhose et al. identified evidence from only one cluster RCT that a software based intervention to identify medications that might contribute to delirium, applied to medication review, reduced delirium in this setting (12-month HR 0.42, CI 0.34 to 0.51) [49]. This supports our results, although we even achieved a tenfold reduction in complicating delirium due to our implemented strategy of individualized and thus more precise patient plus medication review. Hein et al. demonstrated polypharmacy as an independent risk factor for delirium in a population of elderly patients after emergency admission in an observational cohort study and documented a relative risk of 2.33 [50]. In a Dutch comparative retrospective cohort study, a medication review conducted by a clinical pharmacist and a geriatrician for delirium in elderly hospitalized patients showed significant clinical benefit in terms of shortening delirious episode by 6.91 days [51]. The American Geriatrics Society Expert Panel on Postoperative Delirium in Older Adults launched Evidence-Based Recommendation Statements as best practice statements [52]. They clearly recommend avoidance of medications prone to induce delirium postoperatively, which is supported in the Clinical Guidelines for Improving Medication Safety in Older Adults [53] published in 2012 and updated in 2019. Nearly 10 years apart, there does not appear to have been much, if any, change in the overall incidence of delirium described in hospitalized older patients. In the Nature Review, Disease Primers, Delirium 2020 Wilson et al. note that prevention strategies, among others, remain an important challenge worldwide. They also focus on neurotransmitter imbalance due to drug use as a trigger for delirium [54].

To our knowledge, the high effectiveness of our IPM, which always covers quite comprehensive patient aspects, for the prevention of complicating delirium is unique. Based on digital data access and many years of internal medicine and clinical pharmacology experience with more than 38,000 own individual medication reviews since 2011, IPM now takes an average of only 6,5 min per patient. Drug attention in delirium should be mandatory in the preventative approach.

The current German ICD-10 classification 2021 defines delirium in its main heading as a “brain-organic syndrome not caused by alcohol or other psychotropic substances”. However, this contradicts our findings and the current international study situation, which identifies polypharmacy and, for example, drugs acting on the anticholinergic neurotransmitter system as important causes [1, 7, 9, 16, 23]. Therefore, drug-related genesis should always be included as a major classification aspect. This is essential to make the medical profession aware of this relationship, especially because of the highly effective preventative capacity confirmed by our IPM and underestimated so far.

In 2019, the adopted future ICD-11 has been approved by the 72nd World Health Assembly and will enter into force in 2022, and hopefully the ICD revision will soon be implemented in Germany. The ICD-11 “6D70.1 Delirium due to psychoactive substances including medication” in its current version will meet the definitional requirements even better and will not only offer medication-related delirium with intoxication or withdrawal state as a subcategory [55]. This differentiated embedding of medications in ICD-11 is clearly more appropriate because it also considers delirium caused by simple drug use and can thus additionally contribute to future prevention. This brings ICD-11 more in line with WHO patient safety efforts, which is also the aim of our study. Corresponding to our clinical expertise, looking more closely at the effects of different opioids on the various human 5-Hydroxytryptamin (5-HT) transporters and the noradrenaline receptors, Rickli et al. have shown, based on the effects of various opioids on the different human 5-HT transporters and the norepinephrine receptors in vitro, that opioids such as tramadol, fentanyl, tapentadol, oxycodone, methadone, and dextromethorphan can induce serotonin syndrome in clinically relevant numbers of patients through their 5-HT transporter or specific 5-HT_1A_ receptor and/or 5-HT_2A_ receptor interactions respectively [56].

From our experience, this can also mimic delirium and should always be clarified by differential diagnosis. As a consequence of our own supportive findings, we have therefore have proposed to the WHO that serotonin syndrome be included as a separate key in the future ICD-11. Awareness raising and continuous sensitization of physicians to iatrogenic drug-induced and preventable causes is the first and most important step to individual active delirium risk minimization, as shown by the results of the IPM intervention. In addition to predominantly CYP2D6 drug interactions in the context of polypharmacy in our patients, it was the cumulative drug effects and ADR on a pharmacodynamic basis and the need for timely dose adjustment in the presence of organ dysfunction that necessitated medication adjustment in the elderly traumatology patient cohort. Our IPM outcome research provides important evidence for the urgent need to assess polypharmacy for drug and patient safety not only in elderly hospitalized patients, but also in older citizens at increasing risk of delirium, cognitive impairment, and even dementia to accelerate underrepresented promising preventative solutions.

### Strengths and weaknesses

The present study is a retrospective study with all its inherent limitations. The patient records and datasets used were not explicitly designed for the study, and data on outcomes and potential confounding variables may be missing. This is equally true for both, the CG and IG. Difficulties to find an appropriate exposed cohort and comparison group in a retrospective cohort study have to be considered as well. This is a retrospective clinical controlled study involving two sets of data collection conducted by the same investigators for both. Randomly achieved matched groups with high agreement between CG and IG in age, gender, residency, BMI, most diagnoses, and injury patterns supported better comparison of the two groups. The study clearly determines changes in drug distributions and effectiveness of IPM on outcome with clinically relevant association. The study was not biased by knowledge of outcome status, although outcomes had already occurred at the time of recruitment, because both cohorts included samples blinded to outcome. The large differences within overall incidence of delirium in orthopedic and trauma surgery patients in other studies, ranging from 12 to 51% (1) are likely related to very different patient settings and to the fact that delirium is a purely clinical diagnosis and hypoactive or mixed subtypes may be underreported as they also may be in our cohorts. The misinterpretation of delirium as dementia or incipient dementia also needs to be discussed as a cause for the wide variation in incidence. Because of the unavailability of delirium screening assessments and because of the retrospective character of the study, we focused only on delirium complicating hospitalization as a robust and most concise manifestation recorded in the hospital discharge letter. Undocumented, less prominent delirium must also be assumed in our cohort. Because the same senior medical professionals were responsible for patient medical care and IPM throughout the observation period, the recording of complicating delirium and associated factors in the database can be considered fairly accurate and consistent. Confounding was minimized by subsequent adjustment for an extremely broad spectrum of variables as potential co-risk factors. The independent positive association obtained thus excluded a wide-ranged potential confounder spectrum. In this regard, this is the first clinical study to document a strong association of IPM with reduced complicating delirium. The study topic addresses an urgent and demographically increasing public health problem, and IPM emerges as a compelling prevention tool that is still underrepresented.

Not all variables that have been shown to have a significant impact on delirium in other studies could be validated in this study. This is probably due to the different power of the studies compared.

We did not include intraoperative parameters such as operation time and blood loss, which would have been of further interest. But regarding the analysis of an extremely broad range of potential delirium-associated factors, another strength is that the most relevant associated variables identified correspond almost entirely to the prognostic risk factors for the manifestation of delirium recorded in the ICD-10.

With the IPM, a continuity of interdisciplinary cooperation has been established in which the patient-oriented optimization of acute treatment in geriatric traumatology is always in the forefront. The continued focus on avoiding drug-induced risks and individually considering drug interactions and overdosages is likely to have had an additional overarching positive systemic class effect over the years.

The compelling pilot data from our retrospective study of IPM efficacy support the feasibility of designing a future prospective study that includes a larger patient population and comprehensive delirium screening.

## Conclusions

IPM with focus on 6 frontline aspects including reduction of antipsychotics, anticholinergic burden, benzodiazepines, serotonergic opioids, elimination of pharmacokinetic and pharmacodynamic drug interactions and overdosage is highly effective in the prevention of complicating delirium in the elderly trauma patients. Because of the far-reaching consequences of delirium and for the overall patient safety efforts, it should be integrated as an essential and mandatory preventative contribution. The identified delirium-associated factors are consistent with those outlined in ICD-10 and several previous studies and may be helpful if integrated into a consecutively improved, more sophisticated screening scale to identify patients at risk. IPM effectively contributes to the current WHO goal of increasing patient safety in polypharmacy by preventing delirium as a serious complication. Because of its wide-ranging practical relevance, including the upstream outpatient setting, bedside teaching of human medicine students in their final practical year was expanded to include prevention of drug-related risks in the older patients as part of this IPM. For earliest prevention, we have also started to conduct cross-sectoral trainings with great resonance on cognitive risks of polypharmacy including delirium with interprofessional workshops in the outpatient setting throughout Saxony-Anhalt, targeting their primary care physicians, nurses, and pharmacists.

## Data Availability

The datasets generated and analysed for the current study are available from the corresponding author on reasonable request.

## Abbreviations

ACE inhibitors: Angiotensin converting enzyme inibitors
ADR: Adverse Drug Reactions
AV block: Atrioventricular block
BIS formula: Berlin Initiative Study (BIS) eGFR eqs.
BMI: Body mass index = weight (in kg) / height^2 (in m^2^).
CG: Control group
CI: Confidence interval
CNS disorders: Central nervous system disorders
COPD: Chronic obstructive pulmonary disease
CYP2D6: Cytochrome P450 2D6
CYP3A4: Cytochrome P450 3A4
CYP2C19: Cytochrome P450 2C19
DSM: Diagnostic and Statistical Manual of Mental Disorders of the American Psychiatric Association classification system
ECG: Electrocardiogram
FORTA: Fit fOR The Aged
GFR: Glomerular filtration rate
5-HT: 5-Hydroxytryptamin
ICD-classification: International Classification of Diseases
ICU: Intensive care unit
IG: Intervention group
IMC: Intermediate care
IPM: Individual Pharmacotherapy Management
MAI: Medication Appropriateness Index
MCV: Mean corpuscular volume
NSAIDs: Nonsteroidal anti-inflammatory drugs
OR: Odds ratio
Orbis: Hospital information system software
PPI: Proton pump inhibitors
Priscus: Potentially inappropriate medication in the elderly
QT interval: Time from the start of the Q wave to the end of the T wave (measurement on ECG)
SD: Standard deviation
Stopp-Start: Screening Tool of Older Persons’ Prescriptions - Screening Tool to Alert to Right Treatment
UKH: University Hospital Halle (Saale)
WHO: World Health Organization
Würzburger pain drip: Tramadol, metamizole, and metoclopramide
